# The diagnostic value of saccades in movement disorder patients: a practical guide and review

**DOI:** 10.1186/s40734-015-0025-4

**Published:** 2015-10-15

**Authors:** Pichet Termsarasab, Thananan Thammongkolchai, Janet C. Rucker, Steven J. Frucht

**Affiliations:** Movement Disorder Division, Department of Neurology, Icahn School of Medicine at Mount Sinai, 5 East 98th St, New York, 10029 USA; Department of Neurology, University Hospitals Case Medical Center, Cleveland, USA; Division of Neuro-ophthalmology, Department of Neurology, New York University School of Medicine, New York, USA

**Keywords:** Saccade, Eye movement, Ocular motility, Movement disorders

## Abstract

Saccades are rapid eye movements designed to shift the fovea to objects of visual interest. Abnormalities of saccades offer important clues in the diagnosis of a number of movement disorders. In this review, we explore the anatomy of horizontal and vertical saccades, discuss practical aspects of their examination, and review how saccadic abnormalities in hyperkinetic and hypokinetic movement disorders aid in diagnosis, with video demonstration of classic examples. Documentation of the ease of saccade initiation, range of motion and conjugacy of saccades, speed and accuracy of saccades, dynamic saccadic trajectory, and the presence or absence of saccadic intrusions and oscillations are important components of this exam. We also provide a practical algorithm to demonstrate the value of saccades in the differential diagnosis of the movement disorders patient.

**Electronic supplementary material:**

The online version of this article (doi:10.1186/s40734-015-0025-4) contains supplementary material, which is available to authorized users.

## Introduction

Saccades are one of the most useful types of eye movements in the evaluation of the movement disorders patient. The presence of characteristic saccadic abnormalities can be enormously helpful in guiding diagnosis in the outpatient clinic. We present a simplified review the anatomy of horizontal and vertical saccades, discuss practical aspects of their examination, and review saccadic abnormalities in hyperkinetic and hypokinetic movement disorders. Further, we provide an algorithm illustrating the value of saccadic abnormalities in the differential diagnosis of the movement disorders patient. The goal is to provide a practical guide to bedside evaluation of saccades in the context of the movement disorders patient. As such, comprehensive coverage of normal and abnormal ocular motor anatomy and physiology are not included and the reader is referred to comprehensive coverage elsewhere [1, 2].

### Definition of saccades

There are multiple types of eye movements including smooth pursuit, saccades, vestibular and optokinetic reflexes, and vergence [1]. Saccades refer to fast conjugate eye movements that shift the eyes from one target to another, bringing an object of interest into focus on the fovea [3] where visual acuity is highest. Saccades are the fastest eye movements (up to about 500 degrees per second) and they are very brief in duration (typically less then 100 msec) [1]. We will review the anatomy, basic clinical features and examination of normal saccades, and then review movement disorders in which saccadic abnormalities aid in diagnosis.

### Physiology and anatomy of saccades

**Fig. 1:**
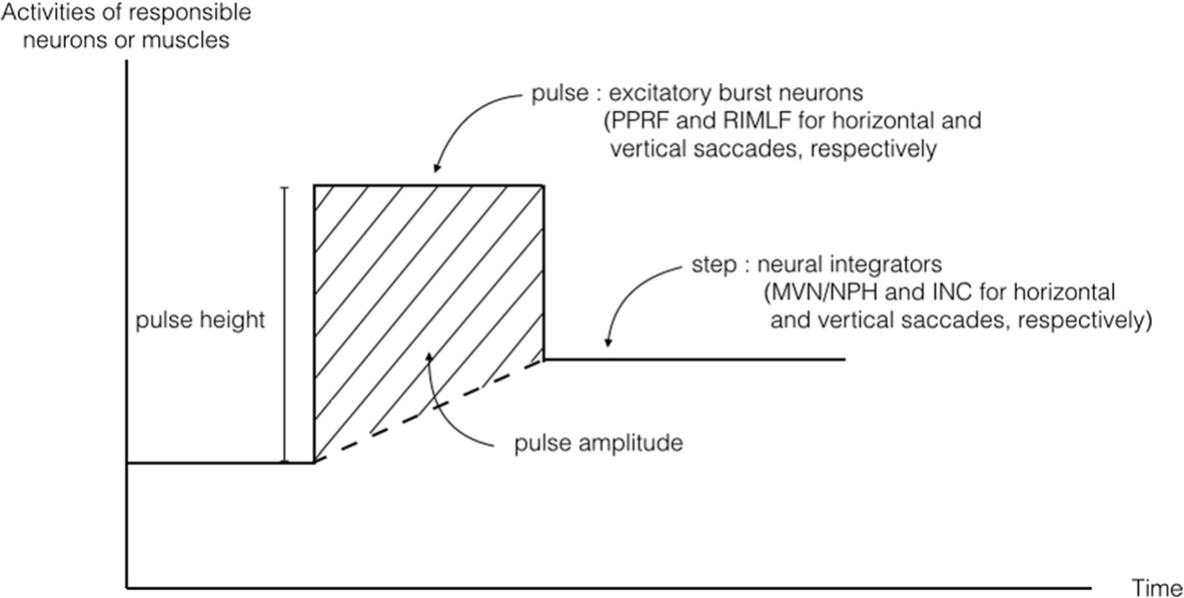
Pulse-step commands of saccades. *X*-axis represents activity of responsible neurons or muscles, and *y*-axis represents time. In initiation of saccades, pulse command is generated by increased firing of excitatory burst neurons, which are the paramedian pontine reticular formation (PPRF) in the pons and the rostral interstitial nucleus of the medial longitudinal fasciculus (RIMLF) in the midbriain, for the horizontal and vertical saccades, respectively. When the eyes reach the new position, step command keeps the eyes with the new level of tonic firing of the responsible neurons, which are the medial vestibular nucleus (MVN) and nucleus prepositus hypoglossi (NPH) in the medulla and the interstitial nucleus of Cajal (INC), for the horizontal and vertical saccades, respectively. Pulse height and area under the curve (or “pulse amplitude) are shown

Initiation of a saccade requires a *“pulse”* of increased firing of *excitatory burst neurons* in the brainstem that results in a high-frequency burst of phasic activity in agonist extraocular muscles [4]. When the eyes reach the new position, a new level of tonic innervation or *“step”* is required by *neural integrators* in order to keep the eyes in this position and overcome the elasticity of the orbital tissues (Figure 1) [5, 6]. *Pulse height* is proportional to the density of the action potential during saccade generation and to peak velocity of saccades, i.e. the smaller the pulse, the slower the peak saccadic velocity. *Pulse amplitude* or *area under the curve* of pulse (pulse height × width) reflects the amplitude of saccades, i.e. abnormally increased area under the curve is related to hypermetric saccades [1]. In the absence of saccade activity, the excitatory burst neurons are inhibited by omnipause neurons. Initiation of the saccadic pulse occurs when the burst neuron is released from its tonic inhibition.

**Fig. 2:**
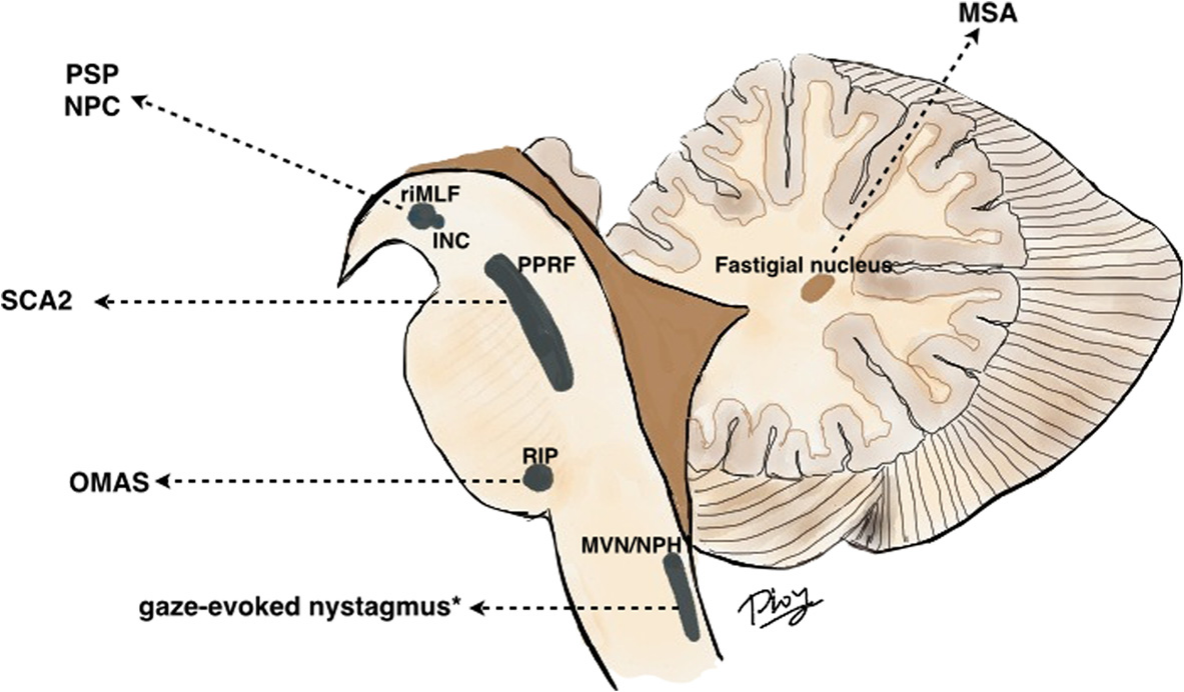
Anatomical substrates for vertical and horizontal saccades. This picture illustrates the brainstem excitatory burst neurons and neural integrators for horizontal and vertical saccades, as well as examples of disorders affecting these structures. For horizontal saccades, excitatory burst neurons are located in the paramedian pontine reticular formation (PPRF) in the pons. The medial vestibular nucleus/nucleus prepositus hypoglossi (MVN/NPH) in the medulla are the horizontal neural integrators. For vertical saccades, excitatory burst neurons are predominantly located in the rostral interstitial nucleus of the medial longitudinal fasciculus (RIMLF), and the interstitial nucleus of Cajal (INC) is the vertical neural integrator. Both of these are in the midbrain. The nucleus raphe interpositus (RIP) in the pons houses the omnipause neurons. *Lesion may not be direct lesion of the MVN/NPH, but may be lesion of the cerebellar feedback circuitry to these structures. Abbreviations: PSP, progressive supranuclear palsy; NPC, Niemann-Pick type C; SCA2, spinocerebellar ataxia type 2; OMAS, opsoclonus-myoclonus ataxia syndrome; MSA, multiple system atrophy; RIMLF, rostral interstitial nucleus of the medial longitudial fasciculus; INC, interstitial nucleus of Cajal; PPRF, paramedian pontine reticular formation; RIP, nucleus raphe interpositus; MVN/NPH, medial vestibular nucleus/nucleus prepositus hypoglossi

These concepts apply to both horizontal and vertical saccades, but neural substrates that control pulse innervation are different than those that control step innervation. For horizontal saccades, excitatory burst neurons are located in the *paramedian pontine reticular formation (PPRF)* in the pons [7], and the neural integrators are the *medial vestibular nucleus (MVN) and nucleus prepositus hypoglossi (NPH)* in the medulla (Fig. 2). For vertical saccades, excitatory burst neurons are in the *rostral interstitial nucleus of the medial longitudinal fasciculus (RIMLF)* in the midbrain, and the neural integrator is the *interstitial nucleus of Cajal (INC)*, also in the midbrain [8, 9]. Omnipause neurons for both horizontal and vertical saccades are located in the *raphe interpositus (RIP)* in the caudal pons. In addition to brainstem saccadic generators, higher level structures including the frontal and parietal lobes, as well as the substantia nigra reticulata and superior colliculi [10], also play critical roles in saccade generation. Full coverage of the anatomy and physiology of these saccadic control centers is beyond the intended scope of this article.

Lesions in each controlling component can lead to different pathologies of saccades (Fig. 2). For example, lesions in PPRF (excitatory burst neurons for horizontal saccades) can give rise to decreased propensity to generate strong bursts (or weak action potential bursts), correlating with slow horizontal saccades, as seen in spinocerebellar ataxia type 2 (SCA2) [11]. Lesions of MVN or NPH or their cerebellar feedback circuitry cause problems holding the eyes in lateral gaze after horizontal saccades, giving a clinical picture of gaze-evoked nystagmus [12, 13]. Lesions in RIMLF can lead to vertical supranuclear gaze palsy in PSP [14] or Niemann-Pick type C (NPC) [15]. Cortical eye field lesions give rise to ocular motor apraxia, such as that seen in Huntington’s disease. Opsoclonus in opsoclonus-myoclonus ataxia syndrome (OMAS) is related to transient impairment in the inhibition through the omnipause neurons in the RIP, though lesions directly in the omnipause neurons cause only saccadic slowing and do not cause opsocolonus [16]. The current mechanisms of such oscillations relate to dysfunction of cerebellar Purkinje cells or to membrane instability and post-saccadic inhibition of burst neurons in the PPRF [17–21].

### How to examine saccades

Saccades can be clinically tested in a *self-paced* or *verbally-guided* manner. For example, one can examine self-paced saccades by asking the patient to make repeated saccades between two visual targets without verbal commands (such as looking quickly back and forth between two pencils placed to the right and left of central fixation), vs. examining verbally-guided saccades by asking a patient to look at the examiner’s nose and then at a target (such as the examiner’s finger) to the left or right of central fixation only upon verbal command. Further the behavior of purely visually-guided saccades without verbal cue to a target that unexpectedly appears in peripheral visual scene (such as a wiggling finger or a shining light) can be assessed. This *reflexive* component of saccades can also be assessed by observation of the fast phases of optokinetic nystagmus (OKN).

When examining saccades, several components deserve careful attention:

1. **Saccade initiation:** Do the eyes promptly generate saccades after commands? Delayed initiation of saccades, also called prolonged latency, is seen in oculomotor apraxia, and in some neurodegenerative disorders such as Huntington’s disease (HD) [22]. Patients with delayed saccadic initiation often employ head thrusts or eye blinks to generate saccades, and these features may be the sole clinical sign indicating a mild defect in saccadic initiation.
2. **Range of motion and conjugacy of saccades:** Do the eyes move to the full gaze extremes up and down and right and left, or is there limitation in the range of motion? Do they move together at the same rate? If there is limited range of motion, the next step is to see if such limitations are present with smooth pursuit and vestibular ocular reflexes (Doll’s eye maneuvers). The hallmark of a supranuclear brainstem saccadic gaze palsy with impaired range of motion, such as that seen with progressive supranuclear palsy (PSP), is a prominent deficit with saccade testing that is improved with smooth pursuit testing and completely overcome with vestibular ocular reflexes.
3. **Speed of saccades:** Do the eyes move slowly during the trajectory from the initial position to the target position? A useful clinical pearl is that one should not be able to follow with one’s own eye the full trajectory of a voluntary saccade, due to the very fast speed of normal saccades. It is important to examine vertical and horizontal saccades independently, as different disorders selectively affect horizontal vs. vertical saccades. Assessment of diagonal saccades (from up and right to down and left, for example) may also be helpful.
4. **Accuracy of saccades:** Do the eyes move accurately to the new target? Are saccades *hypermetric* or *hypometric*? Is there correction of the saccade to target, and is this correction accurate?
5. **Saccadic intrusions or oscillations:** These saccades occur when patients are fixating in the eye primary position, or they may be superimposed during smooth pursuit. Examples include *square wave jerks, macrosaccadic oscillations* and *ocular flutter/opsoclonus*. When square wave jerks occur nearly continuously, they are called square wave oscillations. The main distinguishing features between these movements are their size, whether they move away from and back to midline or oscillate about the midline, their trajectory, and whether or not there is an intersaccadic interval between movements. Square wave jerks consist of a small saccade away from and back to midline with an intersaccadic interval between movements. Macrosaccadic oscillations consist of back-to-back saccades with an intersaccadic interval between movements that oscillate in a crescendo-decrescendo pattern about the midline. Ocular flutter consists of back-to-back saccades without an intersaccadic interval that oscillate about the midline in the horizontal direction only. Opsoclonus is similar to ocular flutter but occurs in all planes (horizontal, vertical, and torsional). Further, one must ask if there are saccadic intrusions during fixation in primary position. Saccadic intrusions are abnormalities of ocular fixation, spontaneous unwanted saccades on regular fixation of a target. A clinical pearl to heighten sensitivity of detection of saccadic intrusions is to have the patient look in lateral extreme gaze and then back to center, as saccadic intrusions are often provoked by gaze shifts. Finally, it should be noted if saccadic intrusions are present during smooth pursuit.

### Saccades in movement disorders

**Fig. 3:**
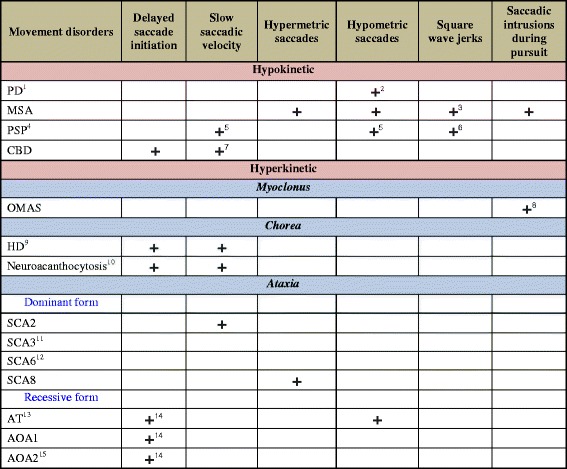
“**+**” indicates presence of the abnormality ^1^Eye movement abnormalities are mostly not detected clinically (without special eye movement recordings) ^2^Especially on self-paced saccades ^3^But not always ^4^Later on, there is limitation of vertical gaze range. Differential diagnosis of vertical supranuclear gaze palsy include corticobasal degeneration (CBD), frontotemporal dementia (FTD), Kufor-Rakeb syndrome (KRS), Niemann-Pick type C (NPC), neuronal intranuclear inclusion disease, Gaucher's disease, and Whipple's disease ^5^In vertical direction; can have round-the-house saccades ^6^Prominent ^7^In some patients with progressive supranuclear (PSP)-like phenotype ^8^Opsoclonus/ocular flutter ^9^Horizontal gaze more affected than vertical gaze, as opposed to PSP. Also has impairment in anti-saccade task ^10^Anecdotally, eye movements tend to be preserved relatively to motor and psychiatric impairment, as opposed to HD ^11^Hypometric vestibulo-ocular reflex ^12^Downbeat, gaze-evoked or rebound nystagmus ^13^Patients can have alternating skew deviation, gaze-evoked or periodic alternating nystagmus; oculocutaneous telangiectasia (not always); elevated alpha-fetoprotein (AFP) ^14^Oculomotor apraxia ^15^Elevated AFP Abbreviations: PD, Parkinson’s disease; MSA, multiple system atrophy; PSP, progressive supranuclear palsy; OMAS, opsoclonus-myoclonus ataxia syndrome; HD, Huntington’s disease; SCA, spinocerebellar ataxia; AT, ataxia-telangiectasia; AOA, ataxia with oculomotor apraxia

We next present abnormalities of saccades as they occur in the clinic (rather than subtle findings from specialized eye movement recording techniques) in hypokinetic and hyperkinetic movement disorders. Main features of saccadic abnormalities of each disorder are also summarized in Fig. 3.

#### Hypokinetic movement disorders

Saccades have an important diagnostic role in differentiating parkinsonian disorders (Video segment 1). Their most obvious utility is in PSP, where vertical supranuclear gaze palsy (VSGP) including slowing of vertical saccades is a crucial diagnostic feature. Multiple system atrophy (MSA) also has saccadic abnormalities as described below, whereas saccades are relatively normal in Parkinson’s disease (PD).

##### Parkinsonian disorders

*Parkinson’s disease (PD).* Hypometric vertical and/or horizontal saccades can sometimes be seen, especially on self-paced saccades [23–25], but these may need special eye movement recording techniques to detect. Clinically (with gross observation at the bedside), saccadic abnormalities are subtle except in severe cases.

*Multiple system atrophy (MSA).* Patients with MSA, especially of the cerebellar type (or MSA-C, olivopontocerebellar atrophy), can have square wave jerks [26] and saccadic dysmetria. Saccadic hypometria [24, 26] and hypermetric saccades (reflecting fastigial nucleus involvement) may also be seen. Saccadic breakdown of smooth pursuit is also common with cerebellar involvement [26], though a very non-specific finding. Although the scope of this article focuses on saccades and does not encompass a comprehensive review of nystagmus and abnormalities of other types of eye movements, it is important to note the presence or absence of gaze-evoked or downbeat nystagmus (DBN), which may only be seen when the patient is placed in a supine position (eg. positioning DBN), in a patient with parkinsonism. If present, these would suggest cerebellar involvement and, thus, might be the main clue to a diagnosis of MSA.

### Progressive supranuclear palsy (PSP) and its mimics

Square wave jerks are common in PSP and are often prominent [27], accompanying the VSGP that defines the illness. In PSP, vertical gaze is typically more affected than horizontal gaze, as the primary pathology is in the midbrain affecting the vertical gaze center or RIMLF. Downgaze may be affected prior to upgaze, but not always. At onset patients may have only slow vertical saccades without limitation of vertical gaze [14], one of the earliest sign of VSGP in PSP. Some patients manifest only progressive vertical gaze slowing and never progress to a limitation of the vertical range of motion in the course of their disease [28]. Careful examination of saccadic velocity is needed to make this diagnosis with confidence [28, 29]. During vertical saccades, especially upgaze, the eyes may follow a curved rather than a linear trajectory, giving the feature of so-called “round-the-house” saccades [24, 30]. This is not specific to PSP, but in fact can be seen in any condition that leads to slowing of vertical saccades relative to horizontal saccades. OKNs are reduced or absent in PSP, vertical more than horizontal [31]. The eyes may appear to follow the OKN stripes, and a common scenario is for only the slow phases of OKN to be generated without any accompanying reflexive saccadic fast phases.

While a VSGP is required for the diagnosis of PSP, a growing list of disorders also include this feature, such as corticobasal degeneration (CBD) or corticobasal syndrome (CBS) [32, 33], frontotemporal dementia [34], Creutzfeldt-Jakob disease [35–39], Kufor-Rakeb syndrome (PARK9 due to *ATP13A2* mutations) [40, 41], Perry syndrome due to *DCTN1* mutations [42], Niemann-Pick type C [15], Whipple’s disease [43], and Gaucher’s disease type 3 (horizontal saccades can also be affected, or even more severe) [44], among others. In Whipple’s disease, in addition to VSGP, patients can have oculomasticatory myorhythmia [45, 46] and pendular convergent-divergent nystagmus [46].

### Corticobasal degeneration (CBD)

Patients with pathologically confirmed CBD or CBS can have VSGP as mentioned above, although a more typical saccadic abnormality is oculomotor apraxia.

#### Hyperkinetic movement disorders

##### Myoclonus

*Opsoclonus-myoclonus ataxia syndrome (OMAS).* Patients with OMAS usually present with vertigo, oscillopsia or ataxia with or without myoclonus [47]. Opsoclonus is a diagnostic feature of this entity (Video segment 2), and can be seen even when eyelids are closed [48]. Opsoclonus is a type of saccadic intrusion/oscillation with spontaneous back-to-back saccades in all trajectories (horizontal, vertical, torsional) without an intersaccadic interval. Both eyes are conjugate during the saccadic intrusions. Ocular flutter refers to a similar movement occurring only in the horizontal trajectory, but there is no functional or clinical difference between opsoclonus and flutter. Opsoclonus and ocular flutter may be post-infectious [49–51] (usually treated with immunomodulating agents such as intravenous steroids, immunoglobulin or Rituximab [52–54]), paraneoplastic [55–58], or due to brainstem encephalitis. In children, an underlying neuroblastoma must be excluded [57, 59, 60].

*Oculopalatal Myoclonus (OPM).* Oculomotor abnormalities in oculopalatal myoclonus are not saccadic (Video segment 2), but the entity merits mention because of its clinical similarity. As in OMAS, eye movement abnormalities in OPM may be more prominent when eyelids are closed. Pendular nystagmus, often with a predominant vertical trajectory occurring at the same frequency as palatal myoclonus (2–3 Hz), is characteristic [61–65].

##### Chorea

*Huntington’s disease (HD).* Oculomotor findings are an important early diagnostic clue in HD patients. The main abnormality is impairment of saccade initiation [22, 66, 67], with or without slowing of saccadic velocity (Video segment 2). Slowed saccadic initiation refers to a delay when a patient is asked to perform saccadic eye movements: the latency from the command to initiation of saccades is long, and vertical saccades are generally more affected than horizontal [68]. Importantly, HD patients also have impairment in anti-saccade tasks: when the examiner confronts the patient, showing a finger on either the left or right side and asks the patient to look at the side contralateral to the appearance of the examiner’s fingers, HD patients make more errors than controls [22, 68–70]. This finding is not, however, pathognomonic for HD and has been seen in many other disorders, including PSP and Dementia with Lewy Bodies.

*Neuroacanthocytosis.* There is no comprehensive description of eye movement abnormalities in neuroacanthocytosis in the literature. Anecdotally, patients can have eye movement abnormalities similar to HD, but saccades tend to be relatively preserved compared to the degree of motor and neuropsychiatric impairment (as opposed to HD where eye movement abnormalities are a cardinal early sign) (Video segment 2). One study showed square-wave jerks, and hypometric horizontal and vertical saccades, as well as limited vertical gaze on eye movement recordings [71].

##### Ataxia

*Spinocerebellar ataxia (SCA).* Saccades are very important diagnostic clues in some types of SCA (Video segment 3). Slowing of saccades, especially on horizontal gaze, is a hallmark clinical feature of spinocerebellar ataxia type 2 (SCA2), first described by Wadia and Swami [11], though slow saccades have also been described in other types, such as SCA1 and SCA7. In our experience, this feature may guide clinicians to pursue initial targeted investigation for the SCA2 gene, instead of ordering the entire ataxia panel. In SCA3, there may be abnormalities of vestibular eye movements, and supranuclear ophthalmoplegia [72]. In SCA6 (typically a pure cerebellar syndrome) and other pure cerebellar SCAs, there may be downbeat and/or gaze-evoked and rebound nystagmus [73]. In SCA8, there are hypermetric saccades [74]. It is worth noting that the horizontal slow saccades of SCA2 and the vestibular deficits of SCA3 are the most suggestive ocular findings of a specific genetic defect. The other SCAs manifest ‘cerebellar eye movements’ including saccadic dysmetria, cerebellar nystagmus, and impaired smooth pursuit in various patterns with substantial overlap in phenotypes. In addition to saccades, examination of optic fundi is also helpful. For example, pigmentary maculopathy is seen in SCA7 [75–78]. Frequent macrosaccadic oscillations are seen in spinocerebellar ataxia with saccadic intrusions (SCASI) [79, 80].

### Recessive cerebellar ataxia

Saccades may be very useful diagnostically in recessive forms of cerebellar ataxia (Video segment 3). In Friedreich’s ataxia, prominent fixation instability may take the form of macrosaccadic oscillations or nearly continuous square wave jerks [81], while interestingly cerebellar atrophy is not seen until the very late stages of the illness [82]. Oculomotor apraxia, an impairment of higher cortical control of eye movements with delayed initiation of saccades and other voluntary eye movements such as smooth pursuit, typically affects horizontal more than vertical gaze. Patients may employ head thrusts or eye blinks to generate saccades, but they are able to generate saccades if given enough time. Oculomotor apraxia can be seen in ataxia with oculomotor apraxia types 1 and 2 (AOA1 and AOA2) and ataxia-telangiectasia (AT) [83–87]. In AT, hypometric saccades, alternating skew deviation, gaze-evoked nystagmus, downbeat nystagmus, upbeat nystagmus, periodic alternating nystagmus, and square wave jerks can be seen [88–90]. In AT and AOA1, after rotating a patient in a chair, there is prolonged post-rotational nystagmus with fast phase (beating) to the direction of vestibulo-ocular reflex (VOR) slow phase [91]. Other clinical and laboratory features are helpful to further distinguish these conditions including careful examination of conjunctiva, palate, pinna or skin in other regions to look for oculocutaneous telangiectasia (seen in AT), elevated alpha-fetoprotein (elevated in AT and AOA2), hypoalbuminemia and hypercholesterolemia (in AOA1 and AOA2).

### Algorithmic approach to movement disorders by utilizing saccades

**Fig. 4:**
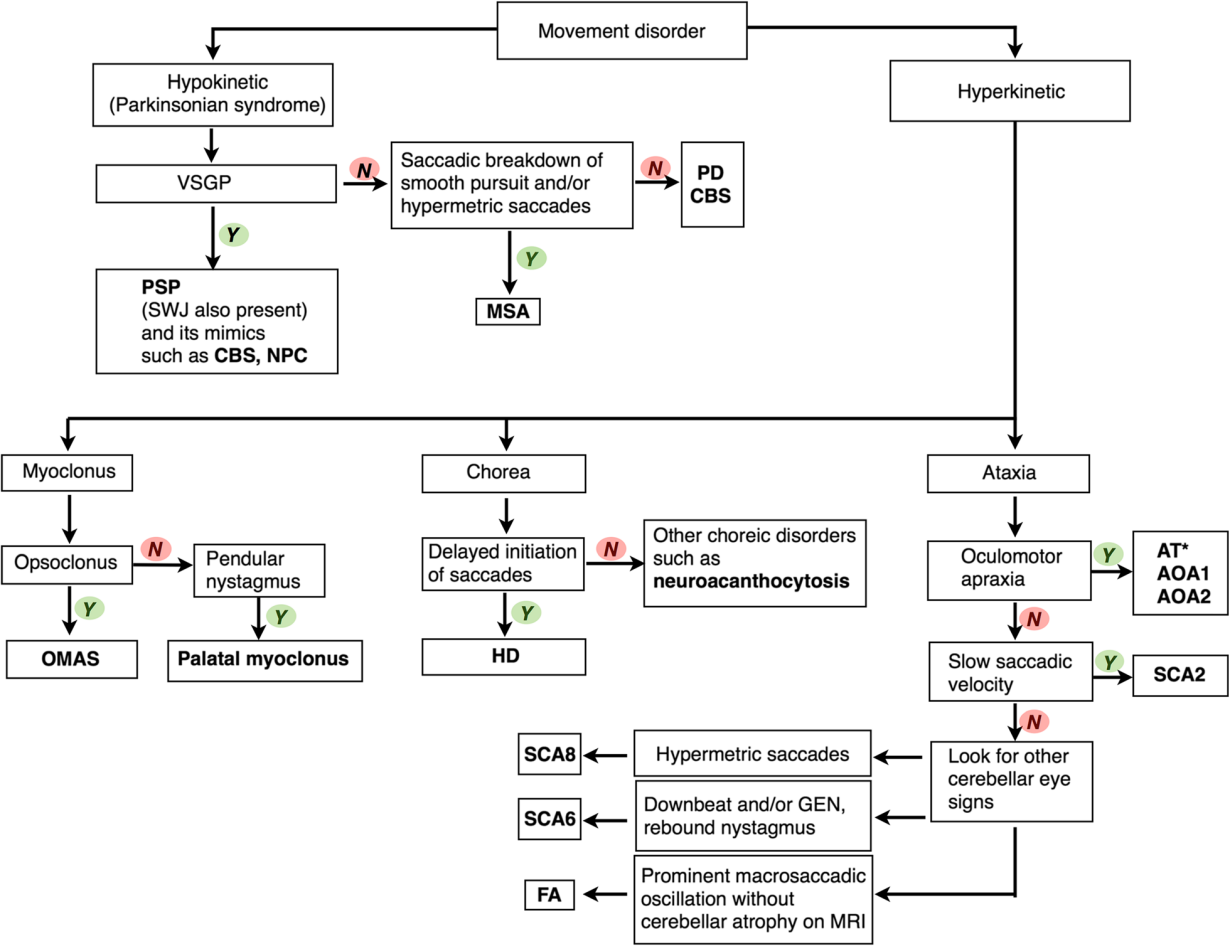
An algorithmic approach to movement disorders utilizing phenomenology and saccades. The approach starts with classifying the patient as hypokinetic or hyperkinetic. Various saccadic abnormalities can help lead to the final diagnosis in each phenomenology. *Cerebellar eye movement abnormalities including downbeat, upbeat, position, gaze-evoked nystagmus and saccadic dysmetria are also common in ataxia-telangiectasia (AT). Abbreviations: AOA1, ataxia with oculomotor apraxia type 1; AOA2, ataxia with oculomotor apraxia type 2; AT, ataxia telangiectasia; CBS, corticobasal syndrome; FA, Friedreich’s ataxia; GEN, gaze-evoked nystagmus; HD, Huntington’s disease; MSA, multiple system atrophy; NPC, Niemann-Pick type C; OMAS, opsoclonus-myoclonus ataxia syndrome; PD, Parkinson’s disease; SCA2, spinocerebellar ataxia type 2; SCA6, spinocerebellar ataxia type 6; SCA8, spinocerebellar ataxia type 8; SWJ, square wave jerks; VSGP, vertical supranuclear gaze palsy

The algorithm displayed in Fig. 4 provides a guide to utilization of saccadic abnormalities in the evaluation of the movement disorders patient. Saccades can be used to pinpoint the diagnosis of many hypokinetic disorder or parkinsonian syndromes, the most obvious of which in this category is PSP and its mimics. Hyperkinetic movement disorders with saccadic abnormalities include myoclonus, chorea or ataxia. However, nothing is absolute and the guide provides an overview of the most common abnormalities and their etiologies and is not comprehensive. We suggest utilization of the algorithm, along with other clinical features including other ocular motor abnormalities not mentioned in the algorithm. In the video segments that follow, outlined in the legend below, various saccadic abnormalities are demonstrated.

## Conclusion

Saccades are a very useful part of the clinical examination in movement disorder patients. Clinicians should be familiar with the appropriate examination of saccades and interpretation of findings of abnormal saccades.

## Consent

Written informed consent was obtained from the patients for publication of all video segments. A copy of the written consent is available for review by the Editor-in-Chief of this journal.

## Additional files

Additional file 1:
**Segment 1. Hypokinetic disorders. Multiple system atrophy (MSA):** This patient with MSA and prominent cerebellar dysfunction demonstrates mild square wave jerks. Saccade attempts on up and downgaze are limited in range. There is hypermetria with overshoot dysmetria in the horizontal direction. Pursuit of a visual target is jerky with saccadic breakdown. **Progressive supranuclear palsy (PSP):** The first patient with PSP demonstrates frequent small-amplitude square wave jerks. When asked to keep his head still and to look up at the ceiling, upgaze saccades are slow and incomplete. When asked to look down at the floor, marked impairment of downgaze saccades is evident. Horizontal saccades are quicker, but also abnormally slowed. The next patient with PSP demonstrates “round the house” saccades due to a vertical supranuclear gaze defect. Horizontal saccades are relatively preserved. Pursuits are relatively normal, though vertical range remains impaired. The next patient with mild PSP demonstrates preserved oculocephalic reflexes in the vertical direction (his mild vertical supranuclear gaze deficit is not shown). The next patient with PSP is shown while an OKN tape is moved vertically outside the field of the camera; vertical OKNs are absent. In contrast, horizontal OKNs are preserved. **PSP mimic:** A young man developed asaccadia and parkinsonism after a difficult repair of an ascending aortic arch aneurysm. He is completely unable to generate vertical saccades, and he recruits his brow muscles when making the attempt. After forced eye closure, his eyes move vertically, but he is then unable to generate any downgaze or even horizontal saccades. In order to overcome this, he fixes on a visually guided target (his cell phone) or his own hand. Pursuit movements tracking the examiner’s hand are also effective. **Corticobasal syndrome (CBS):** This patient with corticobasal syndrome developed severe unilateral limb dystonia and levodopa-unresponsive parkinsonism. Very rare square wave jerks are present. Vertical and horizontal saccades are only mildly impaired. (MP4 37835 kb) Additional file 2:
**Segment 2. Hyperkinetic disorders. Opsoclonus-myoclonus ataxia syndrome:** The first patient, a young woman, developed classic symptoms of opsoclonus-myoclonus ataxia following a viral illness. Paraneoplastic antibody screening was negative. Marked opsoclonus is seen. The second patient initially developed ocular flutter that progressed to opsoclonus over several months. Ocular flutter is present as he moves his eyes horizontally, and bursts of opsoclonus occur with vertical saccades. **Oculopalatal myoclonus:** This brief video clip demonstrates a patient with symptomatic oculopalatal myoclonus. Vertical pendular nystagmus of both eyes occurs at 2–3 Hz frequency. **Subacute sclerosing panencephalitis:** This young man developed rapidly progressive cognitive decline and involuntary movements over several months. Cerebrospinal fluid was positive for measles virus, reflecting reactivation of a childhood infection at the age of two. In this video segment he is in a minimally conscious state, with episodic slow truncal jerks every eight seconds, associated with synchronous forced upward gaze. **Huntington’s disease:** Two patients with genetically confirmed Huntington’s disease (HD) are shown. The first patient with moderately severe HD is able to maintain fixation on a visual target. Upper and lower face chorea is evident. He generates vertical and horizontal saccades with mild delay, however he recruits head thrusts to generate vertical saccades. He is able to generate saccades to visually directed targets, with some impersistence of fixation in vertical gaze. Pursuits are relatively preserved. The second patient, affected with milder HD, demonstrates a delay in saccadic initiation, and recruits head thrusts for horizontal saccades. **Neuroacanthocytosis:** This young man with moderately severe chorea and prominent psychiatric impairment from neuroacanthocytosis demonstrates relatively preserved vertical and horizontal saccades (compared to a similarly affected HD patient). (MP4 28278 kb) Additional file 3:
**Segment 3. Degenerative ataxias. Spinocerebellar ataxia type 2 (SCA2):** This patient with genetically confirmed SCA-2 and a history of mild scanning dysarthria and mild wide-based gait, demonstrates pathognomonic eye findings of SCA-2. Rare square wave jerks are present. She blinks to generate vertical saccades, and vertical saccadic speed is mildly slow. Similar blinks are used to generate horizontal saccades, but horizontal saccades are even slower and more difficult than vertical saccades. **Spinocerebellar ataxia type 3 (SCA3):** This patient with genetically confirmed SCA-3 demonstrates prominent facial dystonia and mild facial masking. Pursuits are full but mildly jerky. When asked to generate saccades to visual target, marked overshoot and correction (ocular dysmetria) is apparent. Saccades are somewhat slow and hypometric. **Freidreich’s ataxia (FA):** This young man with FA demonstrates very prominent macrosaccadic oscillations in primary gaze, which persist in other positions of gaze. **Ataxia telangiectasia (AT):** This young boy with genetically confirmed AT presented with severe ataxia, myoclonus and an elevated alpha-fetoprotein; no telangiectasias were present. There is marked difficulty in initiating and carrying out both vertical and horizontal saccades, consistent with oculomotor apraxia. Pursuits are relatively well preserved. **Ataxia with oculomotor apraxia (AOA):** This young woman developed a slowly progressive ataxia with prominent oculomotor apraxia. Genetic testing for known mutations in AOA-1, AOA-2 and AT was negative. Marked impairment and delay in generating both vertical and horizontal saccades is present. She compensates by using head thrusts to generate saccades. (MP4 38041 kb)
